# The Antibody Receptor Fc Gamma Receptor IIIb Induces Calcium Entry *via* Transient Receptor Potential Melastatin 2 in Human Neutrophils

**DOI:** 10.3389/fimmu.2021.657393

**Published:** 2021-05-13

**Authors:** Omar Rafael Alemán, Nancy Mora, Carlos Rosales

**Affiliations:** Departamento de Inmunología, Instituto de Investigaciones Biomédicas, Universidad Nacional Autónoma de México, Mexico City, Mexico

**Keywords:** neutrophil, Fc gamma receptor, calcium, reactive oxygen species, PKC (protein kinase C), TRPM2 cation channel

## Abstract

Human neutrophils express two unique antibody receptors for IgG, the FcγRIIa and the FcγRIIIb. FcγRIIa contains an immunoreceptor tyrosine-based activation motif (ITAM) sequence within its cytoplasmic tail, which is important for initiating signaling. In contrast, FcγRIIIb is a glycosylphosphatidylinositol (GPI)-linked receptor with no cytoplasmic tail. Although, the initial signaling mechanism for FcγRIIIb remains unknown, it is clear that both receptors are capable of initiating distinct neutrophil cellular functions. For example, FcγRIIa is known to induce an increase in L-selectin expression and efficient phagocytosis, while FcγRIIIb does not promote these responses. In contrast, FcγRIIIb has been reported to induce actin polymerization, activation of β1 integrins, and formation of neutrophils extracellular traps (NET) much more efficiently than FcγRIIa. Another function where these receptors seem to act differently is the increase of cytoplasmic calcium concentration. It has been known for a long time that FcγRIIa induces production of inositol triphosphate (IP_3_) to release calcium from intracellular stores, while FcγRIIIb does not use this phospholipid. Thus, the mechanism for FcγRIIIb-mediated calcium rise remains unknown. Transient Receptor Potential Melastatin 2 (TRPM2) is a calcium permeable channel expressed in many cell types including vascular smooth cells, endothelial cells and leukocytes. TRPM2 can be activated by protein kinase C (PKC) and by oxidative stress. Because we previously found that FcγRIIIb stimulation leading to NET formation involves PKC activation and reactive oxygen species (ROS) production, in this report we explored whether TRPM2 is activated *via* FcγRIIIb and mediates calcium rise in human neutrophils. Calcium rise was monitored after Fcγ receptors were stimulated by specific monoclonal antibodies in Fura-2-loaded neutrophils. The bacterial peptide fMLF and FcγRIIa induced a calcium rise coming initially from internal pools. In contrast, FcγRIIIb caused a calcium rise by inducing calcium entry from the extracellular medium. In addition, in the presence of 2-aminoethoxydiphenyl borate (2-APB) or of clotrimazole, two inhibitors of TRPM2, FcγRIIIb-induced calcium rise was blocked. fMLF- or FcγRIIa-induced calcium rise was not affected by these inhibitors. These data suggest for the first time that FcγRIIIb aggregation activates TRPM2, to induce an increase in cytoplasmic calcium concentration through calcium internalization in human neutrophils.

## introduction

Neutrophils, the most abundant leukocytes in peripheral blood, are considered the first line of defense because these cells arrive first at sites of inflammation or infection (1, 2). Once there, neutrophils display a variety of antimicrobial functions including phagocytosis (3, 4), degranulation, and formation of neutrophil extracellular traps (NET) (5). These functions can be initiated or enhanced by antibodies, in the form of immune complexes, binding to their cognate Fc receptors on the surface of the neutrophil (6). Human neutrophils express constitutively two Fc receptors for IgG, the FcγRIIa (CD32a) and the FcγRIIIb (CD16b). Fc*γ*RIIa contains an ITAM (immunoreceptor tyrosine-based activation motif) sequence in its cytoplasmic tail (7), while Fc*γ*RIIIb is a glycosylphosphatidylinositol (GPI)-linked receptor, lacking a cytoplasmic tail (8). These two neutrophil antibody receptors are not only structurally different but also have been shown to induce unique cellular responses (9). FcγRIIa is the predominant Fcγ receptor mediating phagocytosis, while the contribution of FcγRIIIb to this response is minimal (10). In contrast, FcγRIIIb signaling to the neutrophil nucleus for nuclear factor activation is more efficient than FcγRIIa signaling (11). In addition, FcγRIIIb is the only Fcγ receptor capable of inducing NET formation (12, 13). Although, these reports indicate that each receptor can activate particular cell responses, the signaling capabilities of each receptor are still incompletely understood.

Early reports clearly showed that both neutrophil Fcγ receptors induce an increase in cytoplasmic Ca^2+^ concentration ([Ca^2+^]_i_) (14, 15). However, the mechanism for this response seems to be different for each receptor (15). FcγRIIa signals *via* its ITAM sequence to activate Syk (spleen tyrosine kinase). Syk then phosphorylates enzymes such as PI 3-K (phosphatidylinositol 3-kinase) and PLCγ (phospholipase C*γ*). PLCγ produces inositoltriphosphate (IP_3_), and diacylglycerol (DAG). These second messengers cause calcium release from the endoplasmic reticulum (ER), and activation of PKC (protein kinase C), respectively (8, 16, 17). In sharp contrast, FcγRIIIb-mediated increase in [Ca^2+^]_i_ does not involve IP_3_ formation (15), and the mechanism used by FcγRIIIb to increase [Ca^2+^]_i_ is still unknown. Because FcγRIIIb is a GPI-linked receptor lacking a cytoplasmic tail and with no other subunits known to associate with it, its signaling mechanism is only partially described. Recently, it was reported that the signal pathway, activated by FcγRIIIb leading to NET formation, involves Syk, TAK1 (transforming growth factor-β- activated kinase 1), the MEK (ERK kinase)/ERK (extracellular signal-regulated kinase) cascade, activation of PKC, and activation of the nicotinamide adenine dinucleotide phosphate (NADPH)-oxidase complex, which produce reactive oxygen species (ROS) (18). Therefore, it is possible that FcγRIIIb uses some of these signaling molecules to induce an increase in [Ca^2+^]_i_.

Transient Receptor Potential Melastatin 2 (TRPM2), a nonselective Ca^2+^-permeable membrane cation channel, is highly expressed in myeloid cells (19, 20). This receptor is a member of the TRP family of cation-selective channels that are weakly voltage-sensitive and diversely opened by temperature, mechanical force, pH, and oxidative stress (21, 22). TRPM2 can be opened through directly binding with intracellular adenosine diphosphate ribose (ADPR) (23) and can also be indirectly activated under conditions of oxidative stress (24, 25), acidification (26), and elevated intracellular Ca^2+^ (27). Additionally, in dorsal root ganglion neurons, the activity of TRPM2 is increased by the addition of phorbol 12-myristate 13-acetate (PMA) which leads to activation of PKC (28, 29). Thus, because TRPM2 can be activated by ROS and PKC, two of the second messengers involved in the signaling pathway from FcγRIIIb leading to NET formation (18), it is possible that TRPM2 is used by FcγRIIIb to induce an increase in [Ca^2+^]_i_. In order to test this hypothesis, both FcγRIIa and FcγRIIIb were stimulated by specific monoclonal antibodies, and the increase in [Ca^2+^]_i_ was measured in the presence or absence of pharmacological inhibitors. The neutrophil chemoattractant fMLF (N-formyl-methionil-leucyl-phenylalanine), and also FcγRIIa induced a rapid increase in [Ca^2+^]_i_. FcγRIIIb aggregation also induced an increase in [Ca^2+^]_i_, but this increase was delayed by several seconds. Despite both, FcγRIIa and FcγRIIIb aggregation-induced ROS production, in the presence of diphenyleneiodonium chloride (DPI), an inhibitor of the NADPH-oxidase complex (30), only the FcγRIIIb-induced increase in [Ca^2+^]_i_ was reduced. Similarly, in the presence of Gö6976, an inhibitor of PKC (31, 32), only FcγRIIIb-induced increase in [Ca^2+^]_i_ was reduced. In addition, 2-aminoethoxydiphenyl borate (2-APB) (33–35) and clotrimazole (36–39), inhibitors of TRPM2, reduced FcγRIIIb-induced, but not FcγRIIa-induced increase in [Ca^2+^]_i_. These data show for the first time that FcγRIIIb aggregation activates TRPM2 *via* PKC and ROS for inducing an increase in [Ca^2+^]_i_ in human neutrophils.

## Materials and Methods

### Neutrophils

Neutrophils were purified from heparinized peripheral blood collected from adult healthy volunteers following a protocol previously approved by the Bioethics Committee at Instituto de Investigaciones Biomédicas – Universidad Nacional Autónoma de México (UNAM). Neutrophils were purified exactly as previously described (40).

### Reagents

Bovine serum albumin (BSA) was from F. Hoffmann-La Roche Ltd. (Mannheim, Germany). Gö6976, a PKC inhibitor (catalog number sc-221684); antibiotic (5Z)-7-Oxozeaenol (LLZ 1640-2), a TAK1 inhibitor (catalog number sc-202055); and 2-aminoethoxydiphenyl borate (2-APB), a TRPM2 inhibitor (catalog number sc-201487) were from Santa Cruz Biotechnology (Santa Cruz, CA, USA). 3-(1-methyl-1H-indol-3-yl-methylene)-2-oxo-2,3-dihydro-1H-indole-5-sulfonamide (iSyk), a spleen tyrosine kinase (Syk) inhibitor (catalog number 574711); and fura-2-AM, a calcium indicator (catalog number 344905) were from Calbiochem/EMD Millipore (Billerica, MA, USA). UO126, a MEK inhibitor (catalog number V112A), was from Promega (Madison, WI, USA). Dihydrorhodamine123 (DHR-123) a ROS indicator (catalog number AS-85711), was from Anaspec, Inc (Fremont, CA, USA). The cOmplete™ protease inhibitor cocktail (catalog no. 11697498001) and PhosSTOP™ phosphatase inhibitor cocktail (catalog no. 04906845001) were from Roche Diagnostics (Basel, Switzerland). Diphenyleneiodonium chloride (DPI), an NADPH-oxidase inhibitor (catalog number 300260); phorbol 12-myristate 13-acetate (PMA), a PKC activator (catalog number P8139); N-formyl-Met-Leu-Phe (fMLF), a potent chemotactic peptide (catalog number F6632); pertussis toxin, a G protein-coupled receptor inhibitor (41, 42) (catalog number 516560), clotrimazole, a TRPM2 inhibitor (catalog number C6019), and all other chemicals were from Sigma Aldrich (St. Louis, MO, USA).

The following antibodies were used: anti-human FcγRIIa (CD32a) mAb IV.3 (43) (ATCC^®^ HB-217) was from American Type Culture Collection (Manassas, VA, USA). Anti-human FcγRIIIb (CD16b) mAb 3G8 (44) was donated by Dr. Eric J. Brown (University of California in San Francisco, San Francisco, CA, USA). F(ab’)_2_ goat anti-mouse IgG (catalog number 115-006-003) was from Jackson Immunoresearch Laboratories, Inc. (West Grove, PA, USA).

### Fluorescent Calcium Measurements

Increase of cytosolic calcium concentration was measured by detecting fluorescence changes in neutrophils loaded with Fura 2-AM as previously described (45, 46). Briefly, neutrophils were loaded with 10 μM Fura-2, washed, and resuspended (1.5 x 10^6^ cell/ml) in PBS with 1.5 mM Ca^2+^and 1.5 mM Mg^2+^ and kept on ice. Then, fluorescent changes of a 2-ml stirred neutrophil suspension kept at 37°C were monitored with a LS55 spectrofluorimeter (Perking Elmer; Waltham, MA, USA), using 340 and 380 nm excitation wavelengths and 510 nm emission wavelength. Calcium concentrations were calculated as described by Grynkiewicz et al. (47), using the software FL WinLab 4.00.02 (Perking Elmer; Waltham, MA, USA).

For fMLF stimulation, 3 x 10^6^ Fura-2-loaded neutrophils in 2 ml of PBS with Ca^2+^ and Mg^2+^ were placed in a spectrofluorimeter cuvette and incubated at 37°C for 2 min. After that, fluorescence changes were recorded for 100 s, then 40 μl of 500 nM fMLF were added (final concentration of 10 nM). For FcγR stimulation, 3 x 10^6^ Fura-2-loaded neutrophils were first resuspended in 100 μl PBS containing 10 μg/ml of the corresponding anti-FcγR antibody, and incubated on ice for 20 min. After one wash in cold PBS, neutrophils were resuspended in 2 ml PBS with Ca^2+^and Mg^2+^ and transferred to a spectrofluorimeter cuvette. Fluorescence changes were recorded for 100 s, and then 80 μl of 1.3 mg/ml F(ab’)_2_ goat anti-mouse IgG (final concentration 52 μg/ml) were added to aggregate the receptors.

In some experiments, Fura-2-loaded neutrophils were resuspended in 2 ml PBS containing 1 mM of EGTA and fluorescence changes detected after various stimuli for 300 s. Then, 40 μl of 100 mM CaCl_2_ (final concentration 4 mM) were added, and measurements continued until 450 s. Also, in some experiments, neutrophils were incubated for 30 min before stimulation, with the inhibitors LLZ 1640-2 (10 nM), UO126 (50 μM), iSyk (1 μM), DPI (10 μM), Gö6976 (1 μM), 2-APB (5 μM), clotrimazole (10 μM), or the vehicle dimethyl sulfoxide (DMSO) alone. For Pertussis toxin (2 μg/ml), neutrophils were incubated for 75 min before stimulation.

### Measurement of Reactive Oxygen Species (ROS)

ROS production was assessed by detecting fluoresce changes in neutrophils loaded with dihydrorhodamine 123 (DHR-123). Neutrophils (1 x 10^6^) were resuspended in 100 μl of 15 μM DHR-123 in PBS and incubated for 15 min at 37°C in the dark. Neutrophils were washed with 1 ml PBS, and then resuspended in 100 μl of PBS with the corresponding stimulus as follows. For PMA, neutrophils were resuspended in PBS containing 20 nM of PMA, and then incubated at 37°C in the dark for 45 min. For FcγR stimulation, neutrophils were resuspended in PBS containing 10 μg/ml of the corresponding anti-FcγR antibody, and incubated on ice for 20 min. Next, neutrophils were washed in cold PBS, resuspended in 100 μl of PBS containing 10 μg/ml F(ab’)_2_ goat anti-mouse IgG, and incubated at 37°C in the dark for 45 min. After incubation, 250 μl cold PBS were added and cells kept on ice for 2 min. Finally, neutrophils were centrifuged and resuspended in 1% paraformaldehyde in PBS at 4°C. Neutrophils were stored cold in the dark until analyzed by flow cytometry using an Attune NxT Flow Cytometer (Thermo Fisher Scientific), with the 485 nm (excitation) and 520 nm (emission) filters. Neutrophils were gated by dot-plot analysis and 10,000 cells were acquired per sample. Data analysis was performed using the Flowjo X software (Tree Star Inc., Ashland, OR, USA).

### Statistical Analysis

Quantitative data were expressed as mean ± standard error of mean (SEM). Single variable data were compared by paired-sample Student’s t-tests using the computer program KaleidaGraph^®^ version 4.5.2 for Mac (Synergy Software; Reading, PA, USA). Also, multiple pair-comparisons were performed using Tukey’s test after ordinary one-way analysis of variance (ANOVA). Post hoc differences were considered statistically different at a value p < 0.05.

## Results

### Fcγ Receptors Induce an Increase in Cytoplasmic Ca^2+^ Concentration

**Figure 1:**
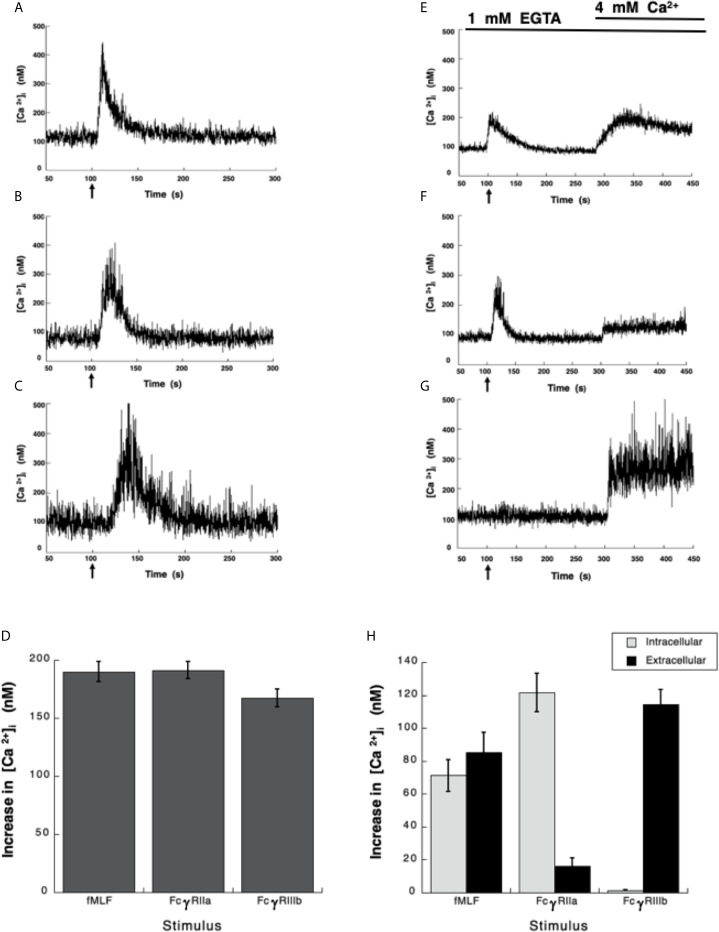
Fcγ receptors induce an increase in [Ca^2+^]_i_. **(A–D)** Fura-2-loaded human neutrophils in PBS with Ca^2+^ and Mg^2+^ were stimulated with 10 nM fMLF **(A)**, or were stimulated by aggregating FcγRIIa on the cell membrane with mAb IV.3 **(B)**, or by aggregating FcγRIIIb on the cell membrane with mAb 3G8 **(C)**. **(E–H)** Fura-2-loaded neutrophils were resuspended in PBS containing 1 mM of EGTA and fluorescence changes detected after stimulating with 10 nM fMLF **(E)**, or by aggregating FcγRIIa **(F)**, or by aggregating FcγRIIIb **(G)**. After 300 seconds, 4 mM Ca^2+^ was added and measurements continued until 450 seconds. Arrow indicates the moment when the stimulus was added. Changes in cytosolic calcium concentration ([Ca^2+^]_i_) were assessed by measuring the variations in fluorescence. Tracings are representative of three experiments with similar results. **(D)** Increments in [Ca^2+^]_i_ were calculated by subtracting the baseline value from the maximum value after stimulation. **(H)** The initial rise in [Ca^2+^]_i_ represents Ca^2+^ from intracellular stores, while the rise in [Ca^2+^]_i_ after addition of 4 mM Ca^2+^ represents Ca^2+^ from extracellular *medium*. Data are mean ± SEM of three independent experiments. Asterisks denote conditions that were statistically different from untreated cells (p < 0.01).

Neutrophils, the most abundant leukocytes in blood, display important functions for controlling infections, inflammation, and immune responses (2, 48). Several of these functions are initiated by antibodies binding to Fc receptors (6). Although, both Fcγ receptors on human neutrophils (FcγRIIa and FcγRIIIb) are known to activate particular cell responses, the signaling capabilities of each receptor are still incompletely understood (9). About 30 years ago, it was already known that both neutrophil Fcγ receptors induce an increase in cytoplasmic Ca^2+^ concentration ([Ca^2+^]_i_) (15), but important differences were noted. FcγRIIa used the second messenger IP_3_ to induce release of Ca^2+^ from an internal pool (15), while the increase in [Ca^2+^]_i_ induced by FcγRIIIb did not involve IP_3_ (15). Despite these early studies the mechanism used by FcγRIIIb to mobilize calcium is still unknown. Thus, in order to further explore the mechanisms of [Ca^2+^]_i_ increase induced by Fcγ receptors, Fura-2-loaded neutrophils were first stimulated with the chemoattractant fMLF. In response to fMLF a rapid almost immediate increase in [Ca^2+^]_i_ was observed (**Figure 1A**). After this rise, the [Ca^2+^]_i_ then returned to basal levels (**Figure 1A**). Similarly, aggregation of the FcγRIIa with specific monoclonal antibodies also induced a rapid increase in [Ca^2+^]_i_ (**Figure 1B**). Also, aggregation of the FcγRIIIb with specific monoclonal antibodies resulted in an increase in [Ca^2+^]_i_ (**Figure 1C**). Although, the magnitude of the [Ca^2+^]_i_ rise was similar with all three stimuli (**Figure 1D**), the FcγRIIIb-mediated increase in [Ca^2+^]_i_ was delayed for about 10 to 20 seconds (**Figure 1C**), suggesting again that the mechanism for calcium mobilization was different between the two Fcγ receptors.

### FcγRIIIb Aggregation Induces an Increase in [Ca^2+^]_i_ *via* Ca^2+^ Entry From Extracellular Medium

It is well established that fMLF-induced increase in [Ca^2+^]_i_ has two components, one initial release of Ca^2+^ from internal stores followed by a subsequent influx of this cation from outside the cell by the mechanism known as store-operated calcium entry (SOCE) (49, 50). Similarly, it is believed that Fcγ receptors also display a similar mechanism for an increase in [Ca^2+^]_i_ (51, 52). Thus, we sought to confirm these ideas by selectively stimulating each Fcγ receptor. Human neutrophils were placed in Ca^2+^-free medium and then stimulated with fMLF. As expected an initial increase in [Ca^2+^]i was observed followed by a gradual return to basal levels around 90 seconds later (**Figure 1E**). At this time and excess of Ca^2+^ was added to the medium. This led to a second increase in [Ca^2+^]_i_, which represents influx of Ca^2+^ from outside the cell (**Figure 1E**). Similarly, in Ca^2+^-free medium aggregation of FcγRIIa induced an increase in [Ca^2+^]_i_ from internal stores that returned to basal levels after about 90 seconds (**Figure 1F**). When Ca^2+^ was restored to the medium a second increase in [Ca^2+^]_i_, was also observed (**Figure 1F**), although this second rise was much smaller. This indicated that similarly to the fMLF, aggregation of FcγRIIa induced a release of Ca^2+^ from internal stores and then an influx of Ca^2+^ from outside the cell. In sharp contrast, in Ca^2+^-free medium aggregation of FcγRIIIb did not induce any increase in [Ca^2+^]_i_ (**Figure 1G**), suggesting that no Ca^2+^ was released from intracellular stores. When Ca^2+^ was restored to the medium an important increase in [Ca^2+^]_i_, was detected (**Figure 1G**). After fMLF stimulation, the magnitude of the [Ca^2+^]_i_ rise from internal stores was very similar to the increase in [Ca^2+^]_i_ from outside the cell (**Figure 1H**). Each part was about half of the total increase in calcium observed in cells kept in Ca^2+^-containing medium. For FcγRIIa stimulation, the magnitude of the [Ca^2+^]_i_ rise from internal stores was similar to the increase in [Ca^2+^]_i_ induced by fMLF (**Figure 1H**). The [Ca^2+^]_i_ rise from extracellular Ca^2+^ was smaller than the [Ca^2+^]_i_ rise from internal stores (**Figure 1H**), suggesting that the major contribution to an increase in [Ca^2+^]_i_ rise after FcγRIIa engagement was from internal stores. In contrast, the FcγRIIIb-mediated increase in [Ca^2+^]_i_ was almost exclusively due to influx of extracellular Ca^2+^ (**Figure 1H**). These data suggested that contrary to other Fcγ receptors, FcγRIIIb induces only an influx of Ca^2+^ from outside the cell.

### FcγRIIIb-Mediated Increase in [Ca^2+^]_i_ Is Independent of TAK1 and MEK

**Figure 2:**
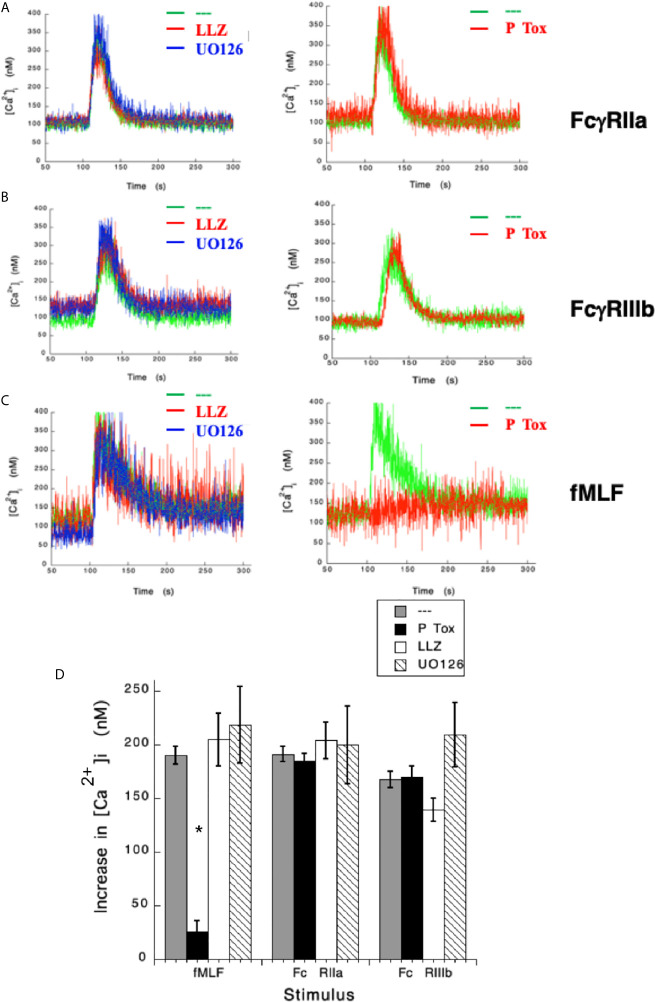
Fcγ receptor-mediated increase in [Ca^2+^]_i_ is independent of TAK1 and MEK. Fura-2-loaded human neutrophils in PBS with Ca^2+^ and Mg^2+^ were left untreated (green line), or treated with 10 nM LLZ 1640-2 (LLZ) a TAK1 inhibitor, or with 50 µM UO126, a MEK inhibitor, or with 2 μg/ml Pertussis toxin (P Tox), before being stimulated by aggregating FcγRIIa **(A)**, or by aggregating FcγRIIIb **(B)**, or with 10 nM fMLF **(C)**. Arrow indicates the moment when the stimulus was added. Changes in cytosolic calcium concentration ([Ca^2+^]_i_) were assessed by measuring the variations in fluorescence as described in material and methods. Tracings are representative of three experiments with similar results. **(D)** Increments in [Ca^2+^]_i_ were calculated by subtracting the baseline value from the maximum value after stimulation. Data are mean ± SEM of three independent experiments. Asterisks (*) denote conditions that were statistically different from untreated cells (p < 0.01).

After having shown that FcγRIIa and FcγRIIIb induce an increase in [Ca^2+^]_i_ very differently, we sought to explore the signaling mechanisms that could help explain these differences. Previously, we have reported that FcγRIIIb signaling activates TAK1 and MEK in human neutrophils (18). Thus, we hypothesized that either TAK1 or MEK were required for the increase in [Ca^2+^]_i_ induced by FcγRIIIb. LLZ 1640-2, a selective TAK1 inhibitor did not affect the increase in [Ca^2+^]_i_ induced by either FcγRIIa (**Figure 2A**) nor FcγRIIIb aggregation (**Figure 2B**). Similarly, UO126, a selective MEK inhibitor did not affect the increase in [Ca^2+^]_i_ induced by either Fcγ receptor (**Figure 2**). Additionally, as expected neither LLZ 1640-2 nor UO126 affected the increase in [Ca^2+^]_i_ induced by fMLF stimulation (**Figure 2C**). However, since the fMLF receptor is a GPCR (53) treatment with Pertussis toxin completely blocked the increase in [Ca^2+^]_i_ (**Figures 2C, D**). In contrast, Pertussis toxin did not affect the increase in [Ca^2+^]_i_ induced by either Fcγ receptor (**Figure 2D**). These data strongly suggested, that TAK1 and MEK were not involved in increasing [Ca^2+^]_i_ after FcγRIIa or FcγRIIIb aggregation in human neutrophils.

### Syk, PKC and NADPH-Oxidase Are Involved in FcγRIIIb-Mediated Increase in [Ca^2+^]_i_

**Figure 3:**
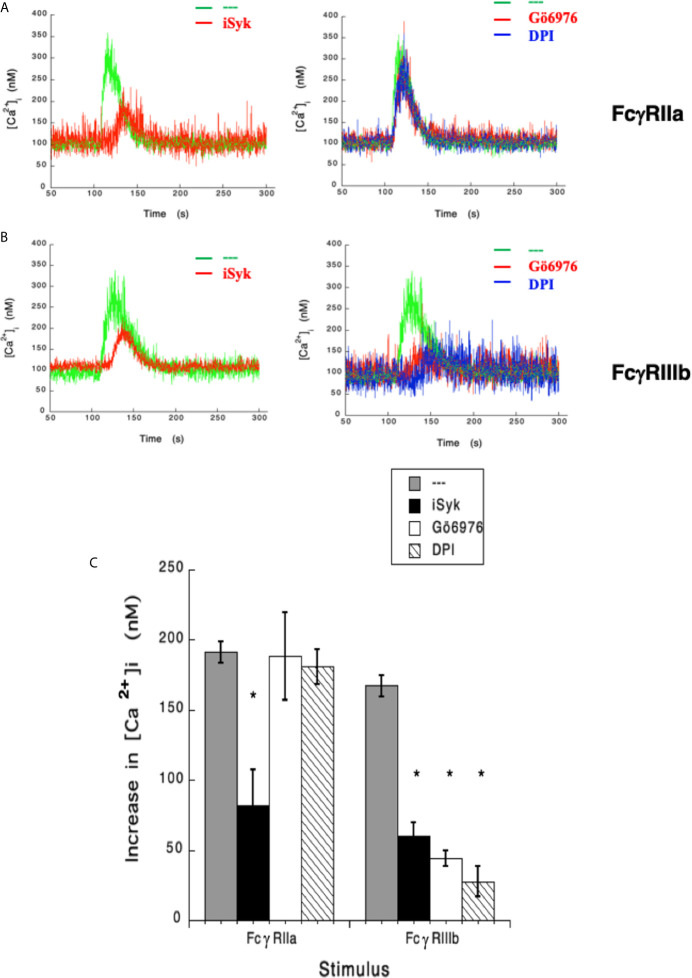
FcγRIIIb-mediated increase in [Ca^2+^]_i_. depends on Syk, PKC and NADPH-oxidase. Fura-2-loaded human neutrophils in PBS with Ca^2+^ and Mg^2+^ were left untreated (green line), or treated with 1 µM iSyk, a Syk inhibitor; 1 µM Gö6976, a PKC inhibitor; 10 µM DPI, a NADPH-oxidase inhibitor, before being stimulated by aggregating FcγRIIa **(A)** or FcγRIIIb **(B)**. Arrow indicates the moment when the stimulus was added. Changes in cytosolic calcium concentration ([Ca^2+^]_i_) were assessed by measuring the variations in fluorescence as described in material and methods. Tracings are representative of three experiments with similar results. **(C)** Increments in [Ca^2+^]_i_ were calculated by subtracting the baseline value from the maximum value after stimulation. Data are mean ± SEM of three independent experiments. Asterisks (*) denote conditions that were statistically different from untreated cells (p < 0.0001).

In the past, we have reported that FcγRIIIb signaling involves Syk, PKC, and NADPH-oxidase (12, 18). Therefore, we explored the possible involvement of these signaling molecules in FcγRIIIb-mediated increase in [Ca^2+^]_i_. Treatment with iSyk, a selective Syk inhibitor, reduced the increase in [Ca^2+^]_i_ induced by either FcγRIIa (**Figure 3A**) or FcγRIIIb aggregation (**Figure 3B**). Additionally, Gö6976 a selective PKC inhibitor blocked the increase in [Ca^2+^]_i_ induced by FcγRIIIb but not by FcγRIIa (**Figure 3**). Similarly, DPI a selective NADPH-oxidase inhibitor reduced the increase in [Ca^2+^]_i_ induced by FcγRIIIb but not by FcγRIIa (**Figure 3**). These data showed that Syk was involved in both FcγRIIa- and FcγRIIIb-mediated increase in [Ca^2+^]_i_, and suggested for the first time that FcγRIIIb signaling, but not FcγRIIa signaling to increase [Ca^2+^]_i_ in human neutrophils involves both PKC and NADPH-oxidase.

### FcγRIIIb-Induced ROS Production Involves Syk and PKC

**Figure 4:**
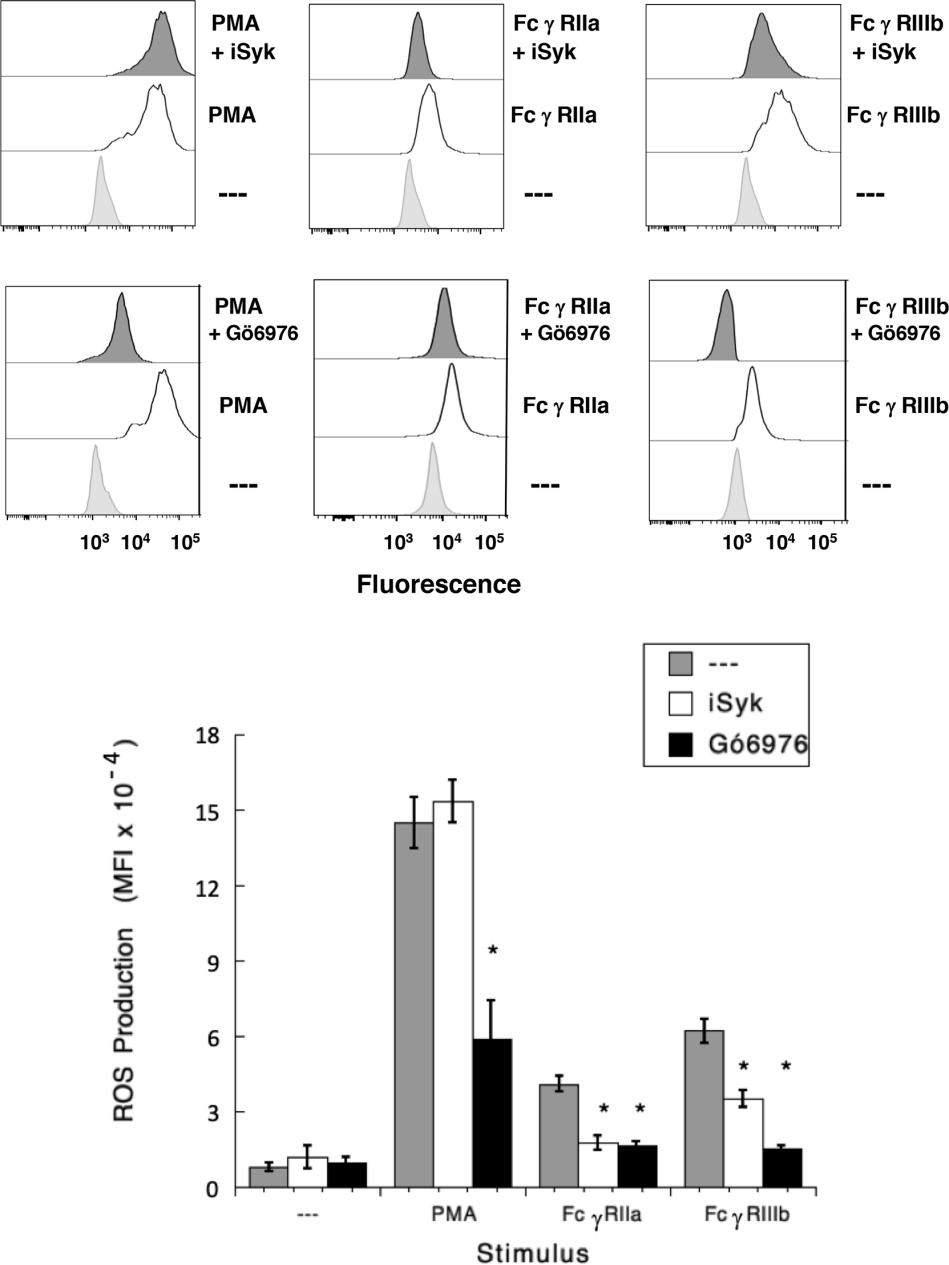
FcγRIIIb-induced ROS production involves Syk and PKC. Reactive Oxygen Species (ROS) production was assessed in dihydrorhodamine 123-loaded neutrophils by detecting fluoresce changes in flow cytometry. (Upper part) Neutrophils were left untreated (light gray), or were stimulated (white) with 20 nM PMA, or by aggregating FcγRIIa, or by aggregating FcγRIIIb. Some neutrophils were previously treated (dark gray) with 1 µM iSyk, a Syk inhibitor; or 1 µM Gö6976, a PKC inhibitor; before being stimulated. (Lower part) Cumulative data (mean ± SEM) of mean fluorescence intensity (MFI) from three independent experiments done in triplicate. Asterisk (*) denote condition that statistically different from untreated cells (*p* < 0.001).

It has also been shown that NAPDH-oxidase is an enzymatic complex responsible for ROS production in human neutrophils (54, 55), and PKC is able to induce NAPDH-oxidase activation (56, 57). Because, FcγRIIIb signaling involves both PKC and NADPH-oxidase activation (12, 18), we explored whether FcγRIIIb requires PKC to induce ROS production. Neutrophils stimulated with phorbol 12-myristate 13-acetate (PMA), a strong PKC activator, produced a robust amount of ROS (**Figure 4**). Similarly, activation of FcγRIIa or FcγRIIIb resulted in significant ROS production, although smaller than the one induced by PMA (**Figure 4**). When cells were pre-treated with iSyk, a selective Syk inhibitor, PMA-induced ROS production was not affected (**Figure 4**). In contrast, iSyk reduced ROS production induced by either FcγRIIa or FcγRIIIb (**Figure 4**). Also, in the presence of Gö6976, a selective PKC inhibitor, PMA-induced ROS production was blocked (**Figure 4**). Similarly, Gö6976 completely prevented ROS production after aggregation of FcγRIIa or FcγRIIIb (**Figure 4**). These data suggested that FcγRIIa and FcγRIIIb both activate Syk, leading to PKC activation and ROS production. However, only in FcγRIIIb signaling these molecules are connected to a rise in [Ca^2+^]_i_ (**Figure 3C**).

### Transient Potential Receptor Melastatin 2 (TRPM 2) Mediates FcγRIIIb-Induced Increase in [Ca^2+^]_i_

**Figure 5:**
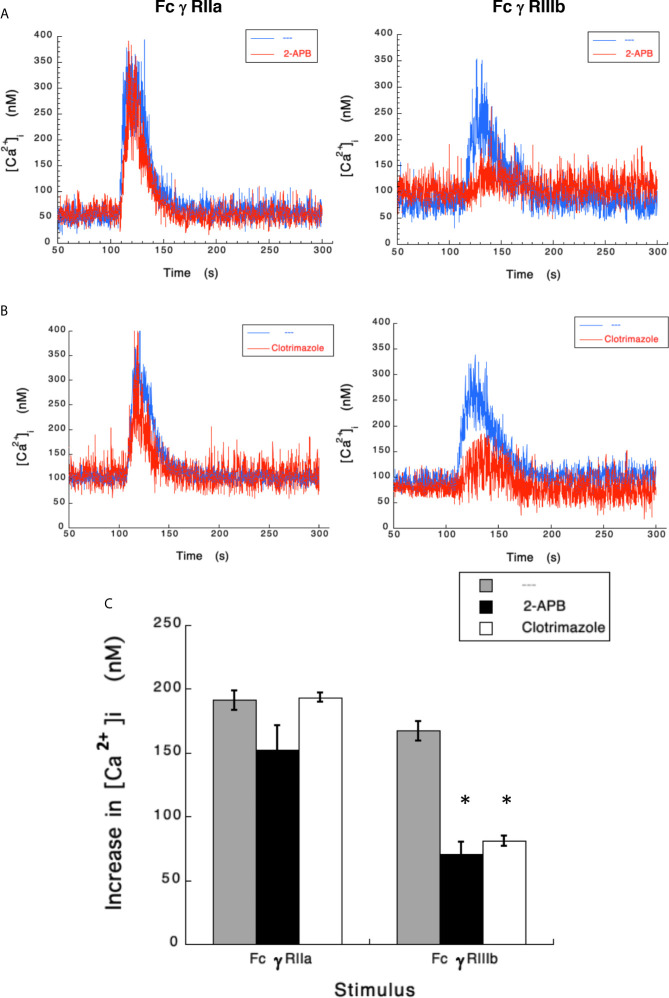
TRPM 2 channel mediates FcγRIIIb-induced increase in [Ca^2+^]_i_. Fura-2-loaded human neutrophils in PBS with Ca^2+^ and Mg^2+^ were left untreated (blue line), or treated (red line) with 5 µM 2-APB **(A)** or with 10 µM clotrimazole **(B)**, two TRPM2 inhibitors, before being stimulated by aggregating FcγRIIa or FcγRIIIb. Changes in cytosolic calcium concentration ([Ca^2+^]_i_) were assessed by measuring the variations in fluorescence as described in material and methods. Tracings are representative of three experiments with similar results. **(C)** Increments in [Ca^2+^]_i_ were calculated by subtracting the baseline value from the maximum value after stimulation. Data are mean ± SEM of three independent experiments. Asterisks (*) denote conditions that were statistically different from untreated cells (p < 0.0008).

**Figure 6:**
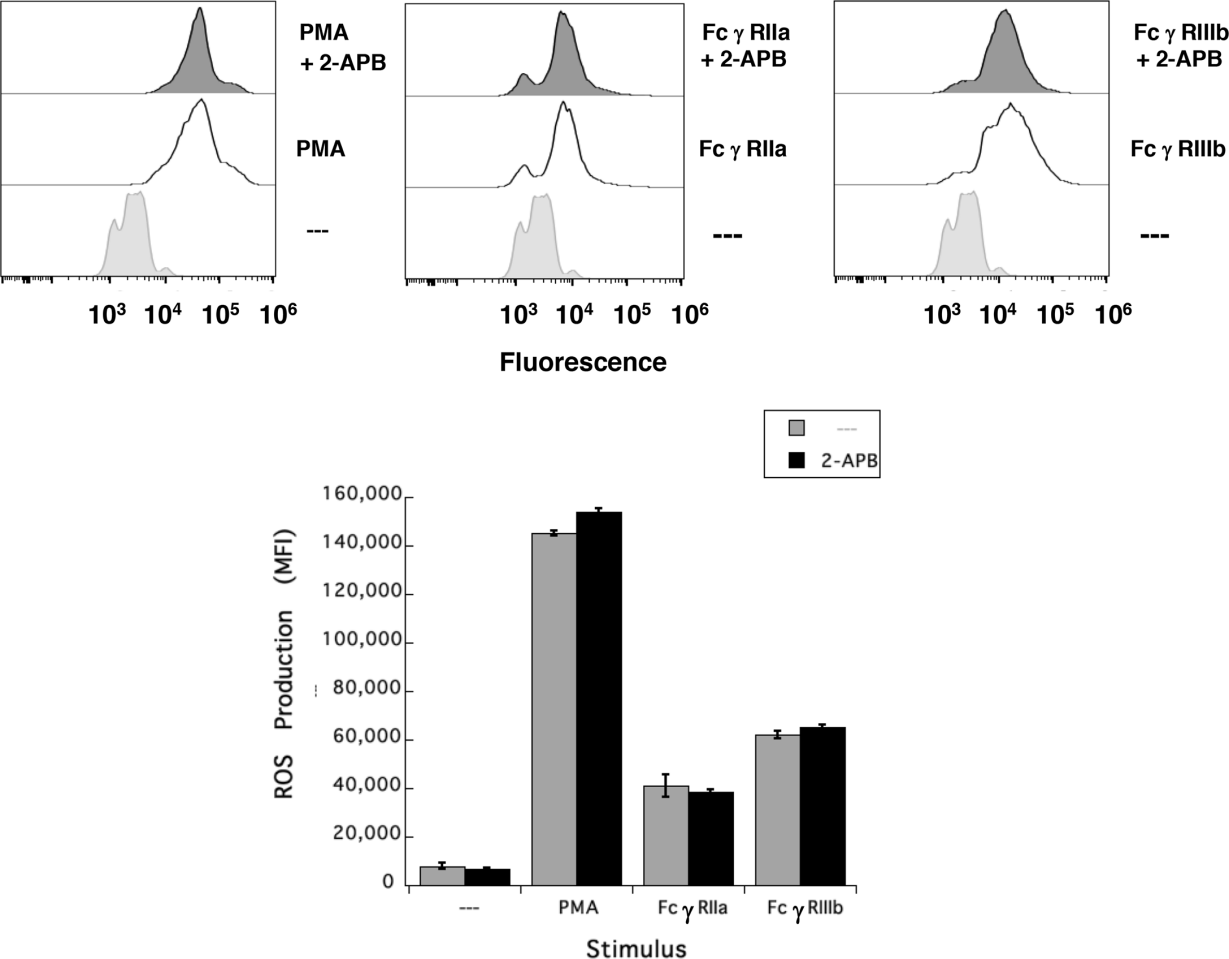
TRPM2 is not required for FcγRIIIb-induced ROS production. Reactive Oxygen Species (ROS) production was assessed in dihydrorhodamine 123-loaded neutrophils by detecting fluoresce changes by flow cytometry. Neutrophils were previously left untreated (—), or were treated with the 5 µM 2-APB, a TRPM2 inhibitor, before being stimulated with 20 nM PMA, or by aggregating FcγRIIa, or by aggregating FcγRIIIb. Data are mean ± SEM of mean fluorescence intensity (MFI) from three independent experiments done in triplicates.

**Figure 7:**
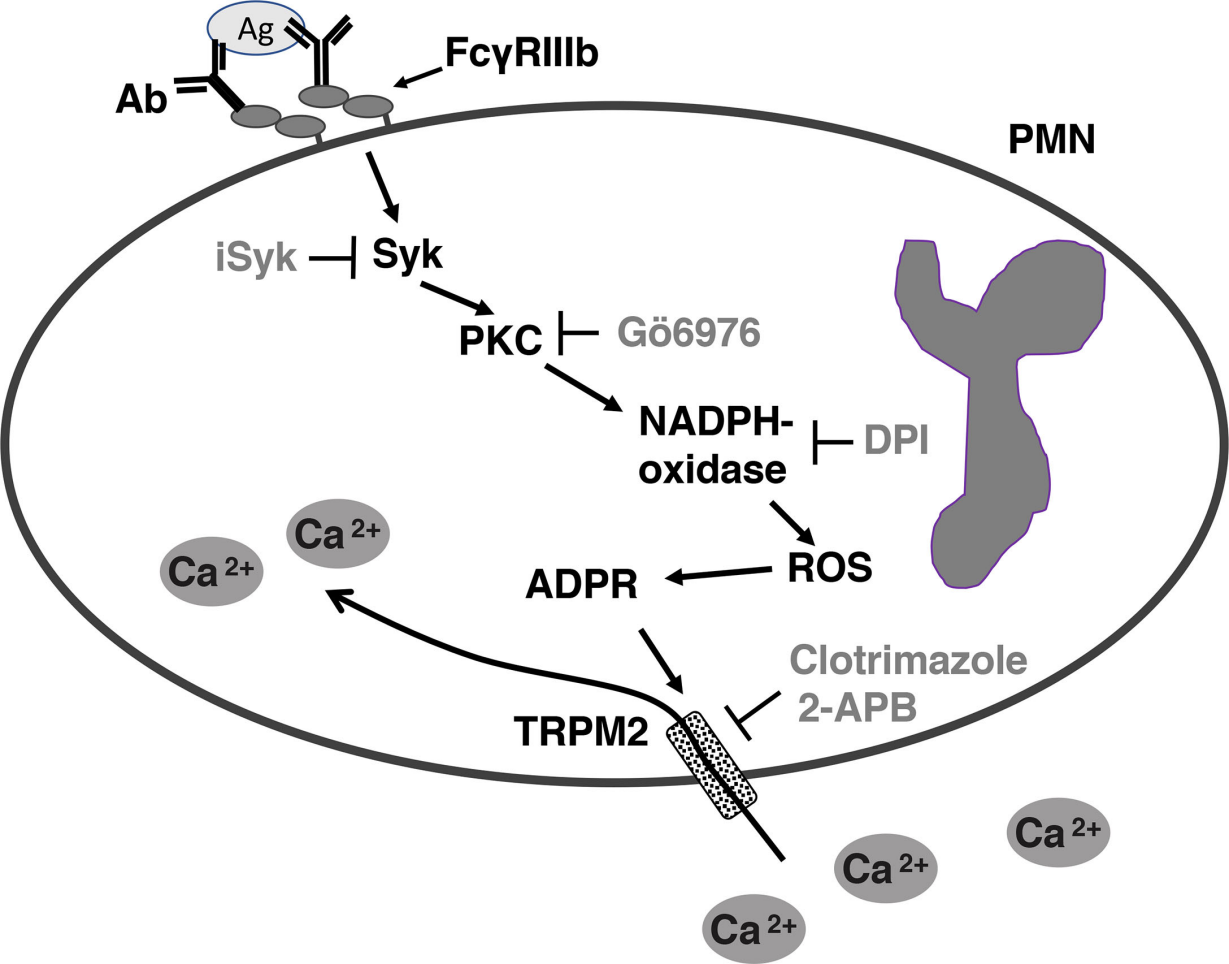
Model for FcγRIIIb-mediated increase in [Ca^2+^]_i_ in human neutrophils (PMN). After aggregation of FcγRIIIb by antibody (Ab)/antigen (Ag) immune complexes, on the plasma membrane of neutrophils (PMN), spleen tyrosine kinase (Syk) gets activated, leading to protein kinase C (PKC) activation. PKC is then required for nicotinamide adenine dinucleotide phosphate oxidase (NADPH-oxidase) activation. NADPH-oxidase, an enzymatic complex assembled on a membrane (not shown), in turn produces reactive oxygen species (ROS), which induce adenosine diphosphate ribose (ADPR) finally leading to activation of transient receptor potential melastatin 2 (TRPM2) channels on the plasma membrane. Activated TRMP2 allow influx of extracellular Ca^2+^ into the cell. iSyk, an inhibitor of Syk; Gö6976, an inhibitor of PKC; DPI, an inhibitor of NADPH-oxidase; 2-APB, an inhibitor of TRPM2; Clotrimazole, another inhibitor of TRPM2.

Results presented above suggested that FcγRIIIb induces PKC activation and ROS production, conducting to activation of a Ca^2+^ channel that allows Ca^2+^ influx into neutrophils. Several ion channels including different TRP family members (19, 21) could be involved in this response (58). In neutrophils several TRP channels were found to be expressed at the mRNA level by RT-PCR, including TRPC6, TRPM2, TRPV1, TRPV2, TRPV5 and TRPV6 (59). Of these channels, TRPM2 was demonstrated to mobilize Ca^2+^ in granulocytes (60), and to be activated by ROS (24, 27, 61, 62) and by PKC (28, 29). Based on these observations, we then explored whether TRPM2 was involved in FcγRIIIb-mediated increase in [Ca^2+^]_i_. Fcγ receptor-mediated increase in [Ca^2+^]_i_ was evaluated in the presence of 2-APB (35) or clotrimazole (37), two different TRPM2 inhibitors. After FcγRIIa aggregation the increase in [Ca^2+^]_i_ was not affected by the presence of 2-APB (**Figure 5**). In contrast, 2-APB efficiently inhibited the FcγRIIIb-mediated increase in [Ca^2+^]_i_ (**Figure 5**). Similarly, the FcγRIIa-mediated [Ca^2+^]_i_ rise was not affected by the presence of clotrimazole (**Figure 5**). However, clotrimazole also efficiently inhibited the FcγRIIIb-mediated increase in [Ca^2+^]_i_ (**Figure 5**). Moreover, 2-APB could not inhibit ROS production induced by PMA (**Figure 6**) nor by FcγRIIa or FcγRIIIb (**Figure 6**). These data positioned production of ROS up-stream of TRPM2 activation and suggested that PKC and ROS are required for FcγRIIIb-induced activation of TRPM2 to promote Ca^2+^ influx in neutrophils (**Figure 7**).

## Discussion

Neutrophils represent the most abundant leukocytes in blood and are considered the first line of defense against invading microorganisms because they are the first leukocytes to arrive at sites of inflammation and infection (63–65). At affected sites neutrophils display several antimicrobial functions (66) including degranulation, production of reactive oxygen species (ROS) (67, 68), phagocytosis (4) and the formation of neutrophil extracellular traps (NET) (5). In addition to these innate immune functions, neutrophils also participate in modulating the adaptive immune response (2). Initiation of these multiple cellular functions involves numerous receptors triggering a myriad of intracellular signaling pathways (52). In many of these pathways, changes in intracellular Ca^2+^ concentration ([Ca^2+^]_i_) are a prerequisite for neutrophil activation (52, 69). However, how changes in [Ca^2+^]_i_ induced by the multitude of receptors expressed on these cells, control neutrophil activation and function remains only partially understood (58, 70, 71). In the case of antibody-mediated neutrophil responses (8, 9), it has been found that both human Fcγ receptors, FcγRIIa and FcγRIIIb, are capable of inducing an increase in [Ca^2+^]_i_ (45, 72, 73). However, important differences on how each receptor mobilizes Ca^2+^ were reported since almost 30 years ago. While, FcγRIIa requires 1,4,5-inositol triphosphate (IP_3_) production for an increase in [Ca^2+^]_i_, the FcγRIIIb-mediated increase in [Ca^2+^]_i_ is independent of IP_3_ (15). Now in this report, we show for the first time, that in human neutrophils stimulation of FcγRIIIb leads to TRPM2 activation to mediate an increase in [Ca^2+^]_i_.

Changes in [Ca^2+^]_i_ are fundamental for the activation process of neutrophils (52, 69), and consequently Ca^2+^ fluxes for neutrophil responses are finely regulated in terms of temporal and spatial organization (58). In resting conditions, [Ca^2+^]_i_ in neutrophils is around 100 nM, a level 10 000-fold lower than the concentration in the extracellular medium (74). Upon stimulation of neutrophils *via* various receptors such as G-protein coupled receptors (GPCRs) (75), integrins (76), or Fcγ receptors (45, 72) there is a rapid increase in [Ca^2+^]_i_ caused by the release of Ca^2+^ from intracellular stores and/or by influx of extracellular Ca^2+^. Engagement of these receptors leads to activation of phospholipase C (PLC). GPCRs mainly activate the PLCβ2 and PLCβ3 (75), while integrins and Fcγ receptors activate PLCγ1 and PLCγ2 (52, 76). PLC in turn act on phosphatidylinositol 4,5 bisphosphate (PIP_2_) to generate diacylglycerol (DAG) and IP_3_. Binding of IP_3_ to its cognate receptor (IP_3_R), which also functions as a nonselective Ca^2+^ channel, localized on the membrane of the endoplasmic reticulum (ER), leads to a release of Ca^2+^ into the cytoplasm (41, 77). The initial rapid release of Ca^2+^ from the ER is followed by influx of Ca^2+^ across the plasma membrane. This influx is induced by the drop in Ca^2+^ levels inside the ER in a process that is known as store-operated calcium entry (SOCE) (78, 79). The mechanism for SOCE involves [Ca^2+^]_i_ sensing proteins such as stromal interaction molecule 1 (STIM1), which migrates from the ER to the plasma membrane when intracellular stores are discharged (78). At the plasma membrane STIM1 gets together with the Ca^2+^ channel protein Orai1 (71, 80), which allows influx of extracellular Ca^2+^. In addition, it has been observed that after receptor stimulation there is also a Ca^2+^ influx that is dependent on receptor occupation by agonists and relatively store independent. This other mechanism of Ca^2+^ entry is known as receptor-operated calcium entry (ROCE) (81, 82). The molecular mechanisms controlling these two components of Ca^2+^ influx are still not resolved (83, 84).

Although the main mechanism for increasing [Ca^2+^]_i_ in neutrophils is primarily mediated *via* IP_3_-dependent Ca^2+^ release from intracellular stores followed by SOCE activation of Orai1 channels (16), there is evidence that additional ion channels *via* ROCE are also involved in calcium influx into these cells (58). This has been clearly demonstrated for fMLF stimulation. After the initial increase in [Ca^2+^]_i_ a second influx of extracellular Ca^2+^is observed. This influx is composed predominantly by Orai1 channels which are selective for Ca^2+^, and also by other non-selective channels that allow entry of both Ca^2+^ and strontium cations (Sr^2+^) (50). In phagocytes, the nature of Ca^2+^ channels mediating ROCE is just beginning to be identified.

An interesting group of nonselective ion channels that may participate in ROCE mechanisms in phagocytes is the superfamily of transient receptor potential (TRP) channels (85). TRP channels are expressed in many cell types and participate in multitude of physiological and pathological processes, such as cell proliferation, differentiation, and death (21). They are particularly important as biosensors of environmental and cellular stimuli including heat, mechanical force (pressure), oxidative (redox) status, and pH (21, 22, 86). The family of TRP channels is divided in six subfamilies: the ankyrin (TRPA), the canonical (TRPC), the melastatin (TRPM), the mucolipin (TRPML), the polycystin (TRPP) and the vanilloid (TRPV) subfamilies. Human and murine neutrophils only express members of the TRPC, TRPM and TRPV subfamilies (58), but their role in ROCE is unclear (81). In mast cells, a ROCE mechanism was described after antigen stimulation. The channel involved allowed influx of both external Ca^2+^ and Sr^2+^ to support degranulation, and was identified as TRPC5 (canonical transient receptor potential channel 5) (87). These reports opened the possibility that TRP channels may contribute to changes in [Ca^2+^]_i_ after Fcγ receptor engagement in human neutrophils.

In phagocytic cells, it is clear that changes in [Ca^2+^]_i_ are important for antibody-mediated cell responses such as phagocytosis (45, 72) and ROS production (67). It is generally accepted that Fcγ receptors activate a signaling cascade that involves Src family kinase-mediated phosphorylation of an ITAM sequence (7) in the cytoplasmic portion of the receptor (or its associated γ chains) (6, 8). The phosphorylated ITAM becomes a docking site for Syk, which in turn activates PLCγ1 to produce IP_3_ (17, 52). This pathway is certainly the one used by FcγRIIa to induce Ca^2+^ release from the ER (15). Then, a SOCE mechanism composed by STIM1 and Orai1 proteins is activated to generate a further influx of Ca^2+^ from outside the cell (16, 71). This influx of extracellular Ca^2+^ was found to be important for the intraphagosomal production of ROS during phagocytosis of opsonized yeast particles (51). We indeed found that selective aggregation of FcγRIIa produces an increase in [Ca^2+^]_i_ resulting from release of Ca^2+^ from intracellular stores (**Figure 1**). For FcγRIIa it is clear that increases in [Ca^2+^]_i_ involve IP_3_-mediated Ca^2+^ release from intracellular stores, followed by a SOCE mechanism that allows Ca^2+^ entry from outside the cell (71, 88).

For FcγRIIIb, elucidating the mechanism for initiating an increase in [Ca^2+^]_i_ has been more complicated, since this receptor is expressed exclusively on human neutrophils, and it is a glycosylphosphatidylinositol (GPI)-linked receptor, lacking transmembrane and cytoplasmic domains (8, 9). Despite that initial steps in signaling for this receptor remain a mystery, it is clear that it can trigger signaling pathways leading to different neutrophil responses including activation of integrins (89), activation of transcription factors (11), and induction of NET formation (12, 13). In addition, FcγRIIIb induces an increase in [Ca^2+^]_i_ (14, 45, 90), which is independent of IP_3_ (15). For a long time, it has been assumed that the initial rise in [Ca^2+^]_i_ must be due to release of Ca^2+^ from intracellular stores by another mechanism that is independent of IP_3_. However, no such mechanism has yet been found. Sphingosine 1 phosphate (S1P), the product of sphingosine kinase (SK) is considered to be a mediator for changes in [Ca^2+^]_i_. In neutrophils, S1P formation was dependent on ER store depletion, and inhibition of SK resulted in a reduction of Ca^2+^ influx (91). Also, in glioblastoma cells it was reported that S1P could activate the TRP channel TRPC1, leading to Ca^2+^ influx (92). In both cases, S1P was found to mediate Ca^2+^ entry into the cells and not release of Ca^2+^ from intracellular stores. Therefore, it is unlikely that S1P mediates release of Ca^2+^ from intracellular stores in neutrophils.

Now, we report that when neutrophils were in Ca^2+^-free medium selective aggregation of FcγRIIIb did not cause any increase in [Ca^2+^]_i_ (**Figure 1**). This result implied that the increase in [Ca^2+^]_i_ was due to an influx of Ca^2+^ from outside the cell. This idea was confirmed when Ca^2+^ was restored in to the medium (**Figure 1**). This finding is in complete agreement with the lack of IP_3_ production when FcγRIIIb is engaged on neutrophils (15). It also pointed to the idea that a membrane ion channel was activated in response to FcγRIIIb aggregation. In previous reports, we described that FcγRIIIb triggers a signaling cascade that involves Syk, TAK1, MEK-ERK for induction of NETosis (18). In addition, we also reported that activation of PKC and production of ROS are important for NET formation (12). Thus, we explored whether these molecules could be involved in FcγRIIIb-mediated increase in [Ca^2+^]_i_. Indeed, we found that PKC and ROS are required for the influx of Ca^2+^ induced by FcγRIIIb (**Figure 3**).

Based on this, we turned our attention to TRP channels which are non-selective ion channels allowing transport of Ca^2+^ (85, 93), and capable (some of them) to sense the redox status in the cell (86, 94). Human neutrophils express the TRP channels: TRPC1, TRPC3, TRPC4, TRPC6, TRPM2, TRPV1, TRPV2, TRPV4, TRPV5, and TRPV6 (58, 59). Among these receptors, only TRPM2 (transient receptor potential melastatin type 2 cation channel; previously also named as TRPC7 or LRPC2) (20, 58) is also known to be activated by PKC (28, 29), and ROS (24, 95). In addition, TRPM2 has been found to be involved in several immune functions, including clearance of bacteria (96–98), NET formation by murine neutrophils (99), activation of NLRP3 inflammasome and secretion of interleukin-1β (100, 101), and dendritic cell maturation and chemotaxis (102). We confirmed that TRPM2 is responsible for FcγRIIIb-mediated rise in [Ca^2+^]_i_ when two inhibitors, 2-APB (33–35, 95) and clotrimazole (36–39) completely blocked FcγRIIIb-mediated rise in [Ca^2+^]_i_ (**Figure 5**). Both inhibitors 2-APB and clotrimazole have been used to block TRPM2 in many cell systems, however they are not specific inhibitors of this channel. Therefore, the possibility remains that other TRP channels may be involved in FcγRIIIb-mediated Ca^2+^ influx. Recently, novel and potentially more specific TRPM2 inhibitors have been reported (103, 104). It would be interesting to use these novel inhibitors to confirm our conclusions. However, we feel confident that TRPM2 is in fact the channel involved because it is the only TRP channel member on human neutrophils that is activated by PKC and ROS. In fact, the mechanism of activation involves adenosine diphosphate ribose (ADPR) binding to the C-terminal domain of the receptor, which presents strong homology to the human nucleotide diphosphate linked moiety X type (Nudix) hydrolase motif 9 (NUDT9) (23, 105). The NADPH-oxidase, like the mitochondrial oxidase, is a molecular complexes vectorially arranged on a membrane such that it accepts an electron from NADPH in the cytosol and transfers it across the membrane reducing oxygen to an oxygen radical (55, 68). The NADPH-oxidase is usually assembled on the phagosomal membrane, to generate ROS into the phagosome, or on the plasma membrane to generate extracellular ROS. Since, oxidative stress induces intracellular accumulation of ADPR it would also be interesting to confirm that aggregation of FcγRIIIb indeed causes an accumulation of ADPR. It is also important to establish whether ADPR is indeed coming from activation of the NADPH-oxidase or from mitochondria in response to oxidative stress. TRPM2 gating requires in addition to ADPR, binding of Ca^2+^ (61, 106, 107). This requirement for full opening of the channel has been very nicely revealed through analysis of cryo-electron microscopy structures of human and zebrafish TRPM2 [reviewed in (108, 109)]. In the case, of FcγRIIIb-mediated TRPM2 activation, we do not know if the basal [Ca^2+^]_i_ is sufficient to support the full opening of the channel. In future experiments, we will eliminate intracellular calcium with BAPTA to further explore the mechanism for Ca^2+^ entry in neutrophils after FcγRIIIb engagement. Also, we will look at the functional consequence of the calcium rise on particular FcRIIIb-mediated neutrophil responses.

The involvement of PKC for inducing activation of TRPM2 comes from indirect studies using PMA (28, 29). In this report, we also demonstrated that indeed PKC is involved in FcγRIIIb-mediated TRPM2 activation using the specific PKC inhibitor Gö6976 (**Figure 3**). However, the particular isoform of PKC that is required for this function remains undetermined. Human neutrophils expressed PKC isoforms from each group of PKC enzymes (110). The inhibitor Gö6976 has specificity for Ca^2+^-dependent PKC isozymes alpha and beta (31). The most likely PKC isoform involved in this response may be PKCβ, since this isoform is an upstream mediator of NADPH-oxidase activation and was reported to be involved in NET formation (111); and we have also reported that FcγRIIIb is the main Fc receptor involved in NETosis (12). Future experiments will determine whether PKCβ is actually required for FcγRIIIb-mediated TRPM2 activation. Tremendous advances have taken place in the field of Ca^2+^ signaling in neutrophils in recent years. However, the literature in this area of research is rather controversial, as human and murine neutrophils and even human HL-60 cells do not always express the same ion channels on their membrane (58, 88). Thus, there is difficulty in integrating the different findings between species, and between cell lines and primary cells. Still, this field has a bright future since there are still many open questions on how calcium signals regulate neutrophil functions (70).

In conclusion, we have found that FcγRIIIb does not induce Ca^2+^ release from intracellular stores but it does activate, *via* PKC and ROS, the TRPM2 channel on the plasma membrane for inducing an influx of extracellular Ca^2+^ into human neutrophils (**Figure 7**).

## Data Availability Statement

The raw data supporting the conclusions of this article will be made available by the authors, without undue reservation.

## Ethics Statement

The studies involving human participants were reviewed and approved by Bioethics Committee at Instituto de Investigaciones Biomédicas – Universidad Nacional Autónoma de México (UNAM). The participants provided their written informed consent to participate in this study.

## Author Contributions

OA performed most of the experiments and analyzed data. NM performed calcium measurements. CR designed the research, mentored other authors, performed statistical analysis, prepared figures, organized the references, and wrote the paper. All authors contributed to the article and approved the submitted version.

## Funding

Research was supported by grant 254434 from Consejo Nacional de Ciencia y Tecnología (CONACyT), Mexico, and by grant PAPIIT IN202520 from Dirección General de Asuntos del Personal Académico, Universidad Nacional Autónoma de México (UNAM), Mexico.

## Conflict of Interest

The authors declare that this research was conducted in the absence of any commercial or financial relationships that could be construed as a potential conflict of interest.
